# NOTA and NODAGA Radionuclide Complexing Agents: Versatile Approaches for Advancements in Radiochemistry

**DOI:** 10.3390/molecules30102095

**Published:** 2025-05-08

**Authors:** Claudia G. Chambers, Jing Wang, Tamer M. Sakr, Yubin Miao, Charles J. Smith

**Affiliations:** 1 Department of Chemistry, University of Missouri, Columbia, MO 65201, USA; cgcgh4@umsystem.edu; 2 Molecular Imaging and Theranostics Center, University of Missouri, Columbia, MO 65201, USA; 3 Research Division, Harry S. Truman Memorial Veterans’ Hospital, Columbia, MO 65201, USA; 4 Stony Brook Cancer Center, Stony Brook University, Stony Brook, NY 11794, USA; jing.wang@stonybrookmedicine.edu; 5 Radioactive Isotopes and Generator Department, Hot Labs Center, Egyptian Atomic Energy Authority, Cairo 13759, Egypt; hafezt@umsystem.edu; 6 Department of Radiology, University of Missouri School of Medicine, Columbia, MO 65201, USA; 7 Department of Radiology, Renaissance School of Medicine, Stony Brook University, Stony Brook, NY 11794, USA; 8 University of Missouri Research Reactor Center, University of Missouri, Columbia, MO 65201, USA

**Keywords:** NOTA, GRPR, SSTR2, MC1R

## Abstract

Effective molecular imaging and targeted cancer therapy rely on receptor-specific targeted delivery systems that are both metabolically stable and kinetically inert for optimal in vivo performance. Until now, no single metal complexing agent has demonstrated the versatility to coordinate metals across the periodic table while maintaining the kinetic inertness required for clinical theranostic applications. Therefore, enhancing the in vivo kinetic stability of radiolabeled, cell-targeting, biologically active compounds remains a critical goal to minimize unintended accumulation of radioactivity in collateral tissues. This review describes the usage of NOTA [NOTA = 1,4,7-triazacyclononane-1,4,7-triacetic acid] and derivatives of NOTA, a metal complexing agent that has been found to have the ability to effectively coordinate with a wide range of radiometals, including metal-radiohalogens, to form stable complexes. This enables the development of new cell-targeting small molecule and peptide conjugates with the potential to resist demetallation in vivo, thereby reducing radionuclide uptake in non-target tissues. Herein, we discuss the design and development of NOTA-based, cell-targeting, small molecules having very high affinity and selectivity for the GRPR (Gastrin-Releasing Peptide Receptor), the SSTR2 (Somatostatin Receptor Subtype 2), and the MC1R (Melanocortin-1) receptors that are present on the surfaces of numerous solid primary human tumors and their metastatic counterparts.

## 1. NOTA/NODAGA Chemistry

Receptor-specific, cell-targeting, small molecule, and peptide-based conjugates for molecular imaging and the treatment of human cancers require metabolically stable and kinetically inert agents to produce high in vivo stability. Traditionally, metal complexing agents, including 1,4,7,10-tetraazacyclododecane-1,4,7,10-tetraacetic acid (DOTA) and 1,4,8,11-tetraazacyclotetradecane-1,4,8,11-tetraacetic acid (TETA), have fulfilled the requirements for the production of highly robust metal complexes with exceptional in vivo stability [1,2]. In fact, DOTA is currently the foundation for producing metabolically stable copper ([^64^Cu^2+^]) [3,4], rare earth ([^177^Lu^3+^]), or rare earth-like ([^68^Ga^3+^]) radiometal complexes for many of the theranostic (therapy and diagnostic) radiopharmaceuticals that have become clinically relevant in the last decade [5,6,7,8,9,10,11].

It is important to also note that these metal complexing agents are not without their own shortcomings. The usage of DOTA or TETA to produce metabolically stable [^64^Cu^2+^] complexes has not always been favorable. The metal complexes, in general, are only moderately stable under in vivo conditions, resulting in demetallation and subsequent accumulation in non-target tissues such as liver. Cross-bridged, cyclam-based ligand frameworks appended to specific biologically active, targeting vectors offer improved kinetic stability to in vivo demetallation and in vivo reactions with various proteins in comparison to DOTA and TETA [12,13,14]. For example, Anderson and Wong have developed cross-bridged macrocyclic chelators that have extraordinary kinetic stability when complexed to [^64^Cu^2+^] [15]. Since that time, they have also shown that phosphonate-based moieties also bind stably to [^64^Cu^2+^] [16]. The cross-bridged chelator (CB-TE1A1P), with one phosphonate pendant arm for the coordination of [^64^Cu^2+^] and one carboxylate arm for conjugation to peptide-based biomolecules, allows for the production of radiocopper complexes that maintain high in vivo stability. However, ligands of this general type still suffer from difficult synthetic protocols, relatively harsh radiolabeling conditions, and renal accumulation and retention of [^64^Cu^2+^] radionuclide [17,18,19].

[^99m^Tc] is a versatile radiometal for use in molecular imaging of human tumors in its own right and still accounts for usage in ~85% of all diagnostic procedures in clinical nuclear medicine facilities [20]. However, much like [^64^Cu^2+^], finding suitable complexing agents that have afforded stability to [^99m^Tc^5+^] metal complexes have not always been useful for the [^99m^Tc^5+^] therapeutic radionuclide surrogates [^186^Re^5+^] and [^188^Re^5+^] [21]. Conventional radiolabeling of biologically active compounds with [^99m^Tc^5+^] or [^186/188^Re^5+^] via N_2_S_2_ or N_3_S donor ligands is an attractive and conventional radiolabeling method [22,23,24]. However, [^99m^Tc^5+^]/[^186/188^Re^5+^]-N_x_S_x_ conjugates often suffer from significant in vivo hydrophobicity, hence have slower clearance from blood serum and non-target tissue. In addition, due to complex differences in the redox potential of [^186^Re^5+^] or [^188^Re^5+^] versus [^99m^Tc^5+^], the in vivo stability of the therapeutic conjugate can oftentimes be compromised by oxidizing agents, resulting in collateral uptake of radioactivity in non-target tissues. Aside from the traditional approach of radiolabeling bioactive molecules (i.e., [^99m^Tc^5+^] or [^186/188^Re^5+^] labeling via N or S chelating donors), an “organometallic” labeling strategy has also been identified, having been pioneered by Jaouen and co-workers [25]. Investigations by Alberto and co-workers led to the development of some remarkable [Tc(I)] and [Re(I)] chemistry [26] and subsequently made the organometallic chemistry and radiolabeling strategies to produce [Tc(I)] and [Re(I)] tricarbonyl complexes containing the [fac-M(CO)_3_] moiety more readily accessible via the Isolink^®^ radiolabeling formulation kit [27]. With the advent of this new organometallic triaqua cation, [^99m^Tc(H_2_O)_3_(CO)_3_]^+^, a new avenue for the successful radiolabeling of bioactive molecules with low-valent [^99m^Tc^1+^]/[^186/188^Re^1+^] was developed [21]. The new [^99m^Tc]Tc(H_2_O)_3_(CO)_3_]^+^ aqua ion has been found to be remarkably stable over a wide range of pH values, presumably due to the low-spin, d^6^ electronic configuration of [Tc(I)]. Furthermore, the lability of the three water molecules coordinated to the [fac-M(CO)_3_] moiety account for the excellent labeling efficiencies with a number of donor groups including amines, thioethers, phosphines, and thiols [28]. Numerous studies have shown bi- and tridentate ligands that comprise primary, secondary, and aromatic amines to be effective chelators for the low-valent metal center, providing the stability necessary for in vivo molecular imaging of human tumor tissue [29,30,31,32,33].

**Figure 1:**
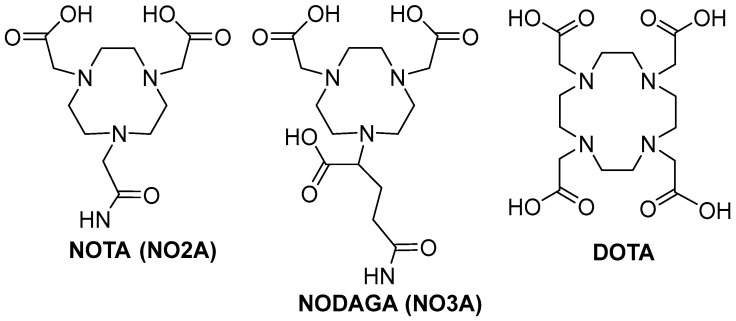
Structural representation of NOTA (1,4,7-triazacyclononane-1,4,7-triacetic acid), NODAGA (1,4,7-triazacyclononane,1-glutaric acid-4,7-acetic acid), and DOTA (1,4,7,10-tetraazacyclododecane-1,4,7,10-tetraacetic acid).

Until now, there has not been a single metal-complexing agent that is considered capable of spanning the periodic table to produce robust, kinetically inert, metal complexes for the development of clinically useful theranostic agents. Thus, there is impetus to continue to improve the in vivo kinetic stability of radiolabeled, cell-targeting, biologically active compounds to reduce the accumulation of radioactivity in collateral tissues including the liver and the kidneys. This review describes the usage of NOTA [NOTA = 1,4,7-triazacyclononane-1,4,7-triacetic acid] and derivatives of NOTA (Figure 1) and how they might fulfill the lofty expectation of a nearly “universal chelating agent”. Meares and co-workers have reported on NOTA, to be used as a bifunctional chelating agent for divalent copper when conjugated to antibodies more than three decades ago [1,2]. However, until the early 2000s, NOTA-based peptide conjugates for [^64^Cu^2+^] and other radiometals for the production of kinetically inert targeting vectors was widely overlooked, and for the most part, largely unexplored [17]. That has changed recently as NOTA has been found to have the capacity to form stable complexes with a plethora of radiometals and even a metal radiohalogen complex [29,34], and, therefore may be a foundation for producing new cell-targeting small molecule and peptide conjugates that overcome demetallation and uptake of radionuclide in non-target tissues. We describe herein the design and development of NOTA-based cell-targeting agents having very high affinity and selectivity for the GRPR (Gastrin-Releasing Peptide Receptor), the SSTR2 (Somatostatin Receptor Subtype 2), and the MC1R (Melanocortin-1) receptors that are expressed on the surfaces of many solid, primary human tumors and their metastatic disease. This report describes, in detail, the synthesis and characterization of novel small molecule and peptide-based conjugates, radiometallation studies to produce metabolically stable and kinetically inert complexes, and detailed in vitro and in vivo investigations in various tumor models. We would like to emphasize, however, that this report is merely a snapshot of the successes of our research groups and only a small group of other investigators [35] around the world that have evaluated radiolabeled, NOTA-based, theranostic, cell-targeting agents and we are excited to finally give this complexing agent the credit that it so strongly deserves.

## 2. Theranostic Isotopes for Usage with NOTA/NODAGA Complexing Agents

In order to produce metabolically stable, kinetically inert radiocomplexes for molecular imaging or targeted radiotherapy (TRT), the radioisotope should be carefully selected. The physical characteristics of the isotope, including half-life, decay mode, and emission profile, are important when choosing a radionuclide for theranostic use. For diagnostic imaging, it is important to select either a positron-emitting radioisotope for positron emission tomography (PET) or a gamma-emitting radionuclide for single-photon emission tomography (SPECT). When choosing a radionuclide for TRT, beta- or alpha-emitting isotopes are essential. Table 1 includes a list of radioisotopes that are known to produce metabolically stable, kinetically inert radiocomplexes with NOTA/NODAGA complexing agents. These isotopes possess the inherent physical characteristics necessary to make them clinically useful for the diagnosis and therapy of human disease.

Copper (II) is considered to be a hard metal center, preferentially complexing to hard donor atoms such as oxygen and nitrogen [17]. [^64^Cu] is a cyclotron-produced radionuclide with a sufficiently long half-life (12.7 h) to be considered readily available for radiopharmaceutical preparation, quality control, drug incorporation, circulation, and patient imaging. [^64^Cu] is produced by irradiation of [^64^Ni] via the (^64^Ni(p,n)^64^Cu) nuclear reaction. [^64^Cu]Cu-labeled radiopharmaceuticals are of primary interest due to the ideal nuclear characteristics of [^64^Cu]: [^64^Cu] (t_1/2_ = 12.7 h; E_β+max_ = 0.65 MeV (17.5%); E_β−max_ = 0.57 MeV (38.5%); electron capture (EC) (44.0%)).

Interest in the [^64^Cu] therapeutic radionuclide surrogate, [^67^Cu], has recently been reinvigorated by the breakthrough in the production of significant quantities of high-specific-activity radiometal. [^67^Cu] is produced in high specific activity (5.55 GBq/μg) by the irradiation of a highly enriched [^68^Zn] target via the (^68^Zn(γ,p)^67^Cu) nuclear reaction. [^67^Cu] has a half-life of 2.58 d, decays by beta emission (E_β_^−^_max_ = 0.562 MeV), and emits two SPECT imaging gamma photons (93 keV, 16% and 185 keV, 49%). High-specific-activity, no-carrier-added [^67^Cu] can be prepared in sufficient quantities for radiotherapy at the National Isotope Development Center [36]. In addition, commercial entities for [^67^Cu] in the United States and Canada are also in the early stages of providing the raw radionuclide to customers. The Idaho State University (Pocatello, ID, USA) Idaho Accelerator Center has recently signed a Product Supply Agreement with Clarity Pharmaceuticals (New South Wales, Australia) for the production of [^67^Cu] for the commercial supply and planned clinical development of new radiotherapeutics.

[^68^Ga] [t_1/2_ = 68 min; E_β+max_ = 1.899 MeV (89%); electron capture (EC) (11%)] is a generator-produced ([^68^Ge]/[^68^Ga]) radionuclide that decays 89% through positron emission (maximum energy of 1.899 MeV, average energy of 0.89 MeV). [^68^Ga^3+^] is considered to be a hard metal center, preferentially complexing to hard donor atoms such as oxygen and nitrogen inherent to chelating agents including DOTA and NOTA [37]. [^68^Ga] has a physical half-life sufficiently long enough for radiopharmaceutical preparation, quality control validation, and PET molecular imaging investigations in patients [38,39]. However, the half-life of [^68^Ga] is not sufficiently long enough for in vivo investigations at later time points. As a result, [^67^Ga] can be used as an imaging substitute due to its extended physical half-life of 78.26 h. [^67^Ga] is produced in a cyclotron by irradiation of [^68^Ni] via the (^68^Ni(p,2n)^67^Ga) nuclear reaction. [^67^Ga] can be used for SPECT scintigraphy, having three gamma emissions (93 keV, 38%; 185 keV, 24%; and 300 keV, 16%). In addition, [^67^Ga] emits Auger and conversion electrons, having the possibility to also be used for therapy.

[^111^In] is a cyclotron-produced radionuclide with a sufficiently long half-life (67.9 h) to be considered readily available for radiopharmaceutical preparation. [^111^In] is produced by irradiation of [^111^Cd] via the (^111^Cd(p,n)^111^In) nuclear reaction and emits two SPECT imaging gamma photons in high abundance (143 keV, 89% and 247 keV, 95%) [40]. [In^3+^] is also considered to be a hard metal center, preferentially complexing to hard donor atoms such as oxygen and nitrogen [40].

[^99m^Tc] is a versatile radiometal for use in the molecular imaging of human tumors due to its ideal physical properties (t_½_ = 6 h and 140 keV gamma emission), on-site availability from a [^99^Mo/^99m^Tc] generator, and diverse radiolabeling chemistry. [^99m^Tc] has proven its utility in nuclear medicine by its use in nearly 85% of all diagnostic procedures in clinical nuclear medicine [41]. [^99m^Tc] is generally considered to be a soft metal center, preferentially complexing to soft donor atoms such as sulfur and phosphorus to produce kinetically inert metal complexes [22].

[^18^F] (t_1/2_ = 110 min; E_β+max_ = 0.635 MeV) is a cyclotron-produced radiohalogen that is nearly ideal for PET because of its physical half-life and low positron energy. As compared to [^68^Ga], with a half-life of 68 min, the 110 min half-life of [^18^F] makes more centralized production and distant distribution possible for [^18^F] radiotracers. Furthermore, having a lower positron energy of [^18^F] (E_β+max_ = 0.635 MeV) as compared to [^68^Ga] (E_β+max_ = 1.899 MeV), a better spatial resolution and, hence, high-quality, high-contrast PET images can potentially be achieved [29,34].

## 3. NOTA/NODAGA Radiometal Complexes of Peptides and Small Molecule Cell-Targeting Agents

### 3.1. Gastrin-Releasing Peptide Receptor (GRPR)-Targeting Radioligands

Bombesin (BBN) is a 14-amino acid peptide having very high affinity for the Gastrin-Releasing Peptide Receptor (GRPR) [42]. The GRPR is found naturally in the nervous system and the gastrointestinal tract, stimulating hormone release. In addition, it is also expressed in very high numbers in various neoplasias including prostate, breast, pancreas, and lung, making it an attractive theranostic target for personalized medicine via targeted oncology [43]. A number of GRPR radioligands have been evaluated preclinically and clinically for the diagnosis and therapy of varying human cancers. A large area of clinical focus using radiolabeled derivatives of BBN or GRPR-targeting peptides continues to be in the area of prostate cancer (PCa) [44]. Prostate cancer cells show elevated levels of GRPR expression, whereas the normal prostate shows only minimal presence of this receptor [44,45,46,47,48].

Early investigations for the development of GRPR-targeting radioligands showed that truncated, agonist peptides had the potential to be used clinically due to their swift internalization into GRPR-expressing tumors. These first-generation GRPR-targeting agents demonstrated prolonged retention within targeted cells due to their inherent internalization properties, driving the impetus for the development of targeted therapeutic agents that take advantage of the cell-killing effects caused by the cross-fire effect of particulate-emitting radionuclides [49,50]. While these first-generation agonists demonstrated satisfactory performance in vivo, the adverse effects on the gastrointestinal system and high pancreatic uptake limited further development.

A shift in the paradigm to produce second-generation GRPR-targeting theranostic radioligands began with the development of SSTR2-targeting antagonists (2006) demonstrating an increase in cell targeting and retention compared to SSTR2 agonists [51]. This shift in strategy began with GRPR-targeting, non-internalizing antagonist radioligands showing highly favorable drug disposition characteristics [52]. Mansi and co-workers prepared RM1 (DOTA-Gly-aminobenzoic acid-D-Phe-Gln-Trp-Ala-Val-Gly-His-Sta-Leu-NH_2_) antagonist radiolabeled with [^111^In] and compared it to the agonist [[^111^In]In-AMBA] radiotracer [53]. [[^111^In]In-RM1] demonstrated superior cell-targeting abilities when compared to [[^111^In]In-AMBA], propelling further investigations with [[^68^Ga]Ga-RM1]. [[^177^Lu]Lu-RM2], where RM2 = ((DOTA-4-amino-1-carboxymethyl-piperidine)-D-Phe-Gln-Trp-Ala-Val-Gly-His-Sta-Leu-NH_2_), is currently being evaluated clinically by the United States Food and Drug Administration (FDA) for the treatment of patients with metastatic, castration-resistant, prostate cancer [54,55]. Studies in four patients who have received [[^68^Ga]Ga-RM2] for PET/CT and [[^177^Lu]Lu-RM2] for therapy showed high tumor uptake, efficient metabolic clearance, minimal retention in collateral tissues, and no observable side effects. In addition, GRPR-positive cells did not show changes in the levels of expression upon being exposed to up to 10 Gy of external beam radiation [56].

**Figure 2:**
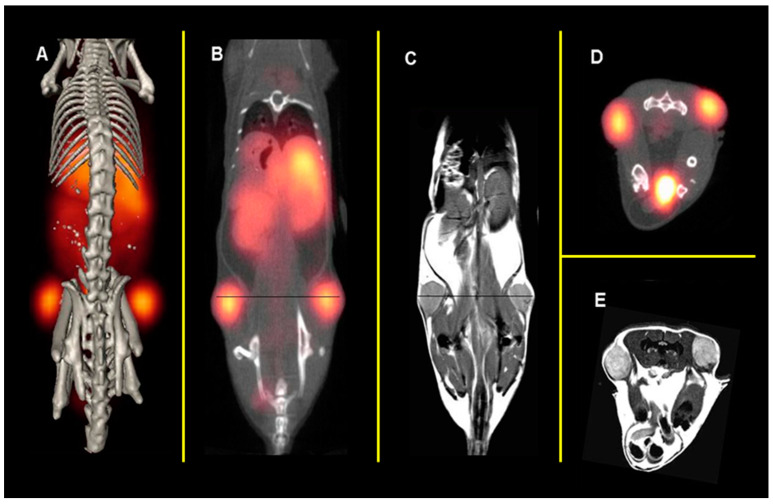
Whole-body, small animal PET/CT of PC-3 tumor-bearing mice using [[^64^Cu]-NOTA-8-Aoc-BBN(7-14)NH_2_] radiotracer. (**A**) Small animal coronal PET/CT; (**B**) small animal coronal PET/CT slice; (**C**) small animal coronal MRI; (**D**) small animal transaxial PET/CT slice; (**E**) small animal transaxial MRI. Figure adapted and modified from reference [17].

**Figure 3:**
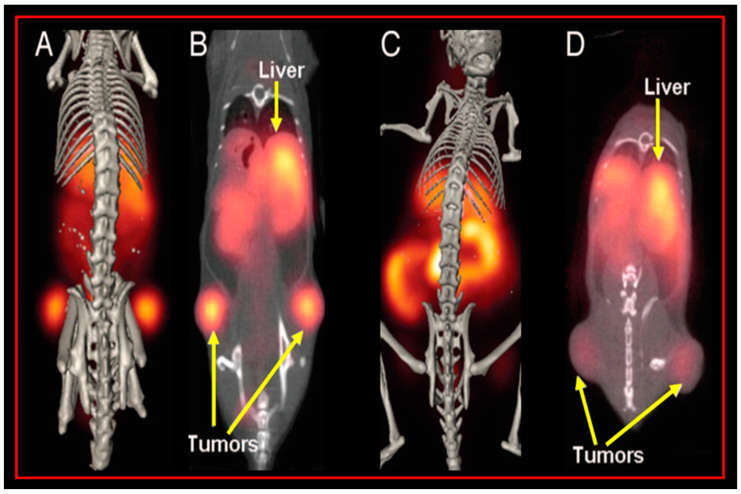
Whole-body, small animal PET/CT of PC-3 tumor-bearing mice comparing [[^64^Cu]-NOTA-8-Aoc-BBN(7-14)NH_2_] (**A**,**B**) and [[^64^Cu]-DOTA-8-Aoc-BBN(7-14)NH_2_] (**C**,**D**) radiotracers. Figure adapted and modified from reference [17].

NOTA has the capacity to form stable complexes with [Cu^2+^] as well as with a host of other di- and trivalent metal centers [57,58]. To our knowledge, our research group in 2007 was the first to evaluate the capacity of NOTA to produce metabolically stable, peptide-based, radioconjugates targeting the GRPR when radiolabeled with [^64^Cu] [17]. In this study, [NOTA-X-BBN(7-14)NH_2_] conjugates were prepared, where X = b-Ala (beta-alanine), 5-Ava (5-aminovaleric acid), 8-Aoc (8-aminooctanoic acid), GGG (glycylglycylglycine), and SSS (serylserylserine) modifiers. The inhibitory concentration 50% (IC_50_) of [NOTA-8-Aoc-BBN(7-14)NH_2_] was determined by a competitive displacement cell-binding assay in PC-3 human prostate cancer cells using [[^125^I]I-[Tyr^4^]-Bombesin] as the displacement radioligand. An IC_50_ of 3.1 ± 0.5 nM for [NOTA-8-Aoc-BBN] was obtained, demonstrating very high binding affinity of the conjugate for the GRPR. [[^64^Cu]Cu-NOTA-X-BBN] conjugates were prepared by the reaction of [[^64^Cu]CuCl_2_] with peptides in buffered aqueous solution. In vivo studies for [[^64^Cu]Cu-NOTA-8-Aoc-BBN(7-14)NH_2_] in tumor-bearing PC-3 mice showed high uptake in tumors, also indicating high affinity of the radiotracer for the GRPR. The accumulation of [[^64^Cu]Cu-NOTA-8-Aoc-BBN(7-14)NH_2_] in tumors was 3.58 ± 0.70% ID/g at 1 h p.i. Minimal accumulation of radioactivity in liver tissue (1.58 ± 0.40% ID/g, 1 h p.i.) is indicative of rapid renal-urinary excretion and suggests very high in vivo kinetic stability for [[^64^Cu]Cu-NOTA-8-Aoc-BBN(7-14)NH_2_] with little or no dissociation of [^64^Cu^2+^] from the [^64^Cu]Cu-NOTA complex. Kidney accumulation at 1 h p.i. was 3.79 ± 1.09% ID/g. Small animal molecular imaging studies in GRPR-expressing PC-3 tumors in SCID mice produced high-quality, high-contrast, PET images (Figure 2). In addition, when compared to a similar DOTA construct of the very same radiotracer structure (Figure 3), [[^64^Cu]Cu-NOTA-8-Aoc-BBN(7-14)NH_2_] showed clearly superior results in vivo. When evaluated in T-47D human breast cancer cells, [[^64^Cu]Cu-NOTA-8-Aoc-BBN(7-14)NH_2_] showed uptake in xenografted tumors of 2.27 ± 0.08, 1.35 ± 0.14, and 0.28 ± 0.07% ID/g at 1, 4, and 24 h p.i., respectively. Small animal molecular-imaging investigations in GRPR-expressing T-47D tumors in SCID mice produced high-quality, high-contrast, PET images [18].

**Figure 4:**
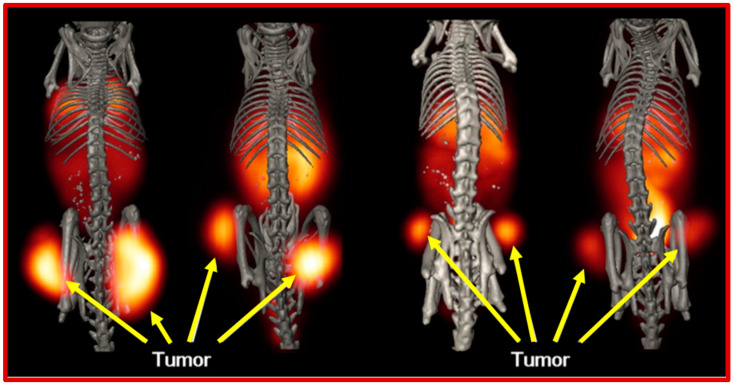
Structure–activity relationship study comparing [[^64^Cu]-NOTA-X-BBN(7-14)NH_2_] radiotracers in PC-3 tumor-bearing mice where X = AMBA *p*-aminobenzoic acid, 6-Ahx 6-aminohexanoic acid, 8-Aoc 8-aminooctanoic acid, and 9-Anc 9-aminonanoic acid (left to right). Figure adapted and modified from reference [59].

A complete structure–activity relationship study for [[^64^Cu]Cu-NOTA-X-(7-14)NH_2_] that included additional pharmacokinetic-modifying linkers including *para*-aminobenzoic acid (AMBA) was promising, showing high uptake and retention in tumors [50]. [[^64^Cu]Cu-NOTA-AMBA-BBN(7-14)NH_2_] showed uptake in xenografted tumors of 6.05 ± 1.15, 3.66 ± 0.54, and 2.54 ± 0.48% ID/g at 1, 4, and 24 h p.i., respectively. Whole-body, small animal PET images showed very high tumor uptake in tumor-bearing PC-3 with little or no accumulation of radiotracer in surrounding, collateral tissues at 18 h p.i. (Figure 4) [59].

Building on previous investigations that showed the successes of using NOTA-based complexing agents to produce preclinically useful [^64^Cu] radiotracers that targeted the GRPR, Makris and co-workers used NOTA and NODAGA (1,4,7-triazacyclononane,1-glutaric acid-4,7-acetic acid) to target the GRPR using the GRPR peptide antagonist (DPhe-Gln-Trp-Ala-Val-Gly-His-Sta-Leu-NH2). The [^64^Cu]Cu-NOTA/NODAGA constructs were linked to the GRPR-targeting motif via a 6-aminohexanoic acid pharmacokinetic modifier [60]. Both derivatives showed high in vitro stability (≥98%, PBS, pH = 7.4, 37 °C, 24 h post-incubation) and high GRPR binding affinity with IC_50_s < 3 nM. In vivo biodistributions were conducted in PC-3 tumor-bearing ICR SCID mice. Both derivatives demonstrated high tumor uptake/accumulation (NOTA: 4.99 ± 1.40% ID/g; NODAGA: 4.07 ± 0.51% ID/g) at 1 h p.i. [60]. NOTA and NODAGA derivatives were excreted via the renal-urinary pathway and showed limited non-target accumulation except for in the pancreas, as it has a high GRPR physiological concentration. Blocking studies confirmed the GRPR mediated the uptake of both derivatives as tumor uptakes were nearly diminished [60]. Small animal PET images in PC-3 tumor-bearing mice validated the biodistribution investigations where high-quality, high-contrast images were obtained. From these preclinical studies, it appears that NOTA is superior to NODAGA for the production of radiotracer and therefore, would be a useful radiotracer for further clinical investigations.

**Figure 5:**
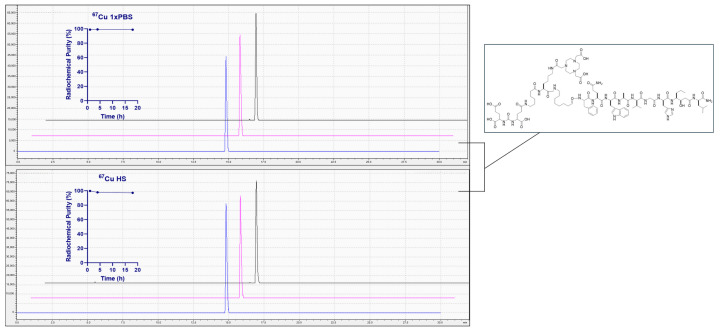
Stability assays of [DUPA-6-Ahx-([^67^Cu]Cu-NOTA)-8Aoc-BBN ANT] evaluated at 1 (blue), 4 (pink), and 18 h (black) time points in phosphate-buffered saline (PBS) (21 °C) or human serum (37 °C).

Thus far, there has been limited research on the development of [^67^Cu]Cu-NOTA radiotracers targeting the GRPR. In fact, an extensive search of the literature produced zero findings of peer-reviewed scholarship for conjugates of this type. Preliminary findings from our very own research group showed effective radiolabeling of [^67^Cu] to both NOTA and NODAGA conjugates of a bivalent PSMA-/GRPR- targeting heterodimer that has been extensively studied both in vitro and in vivo [61]. Both the [DUPA-6-Ahx-(NOTA)-8-Aoc-BBN ANT] and [DUPA-6-Ahx-(NODAGA)-8Aoc-BBN ANT] conjugates had high specific activity of 430 µCi/nmol (≥97%) and no notable degradation for up to 18 h in PBS or human serum (Figure 5). Preclinical validation of these novel radioconjugates in animal models are currently underway.

The ability to target either GRPR or α_v_β_3_ biomarkers simultaneously via a heterodimeric targeting ligand has provided a new avenue to investigate the dual targeting capacity of bivalent radioligands for improved in vivo PET images of specific human cancers. Integrins are cell-surface transmembrane glycoproteins that exist as heterodimers. α_v_β_3_ and α_v_β_5_ integrin subtypes are expressed on the endothelial cells of tumor neovasculature during angiogenesis, and form the basis of investigations for molecular imaging of angiogenesis and tumor formation in vivo. The α_v_β_3_ integrin is known to be expressed in very high numbers in many tumor cell types including lung carcinomas, osteosarcomas, breast cancer, and glioblastomas. α_v_β_3_ is also over-expressed on the surfaces and at different stages of some prostate tumors [62,63].

**Figure 6:**
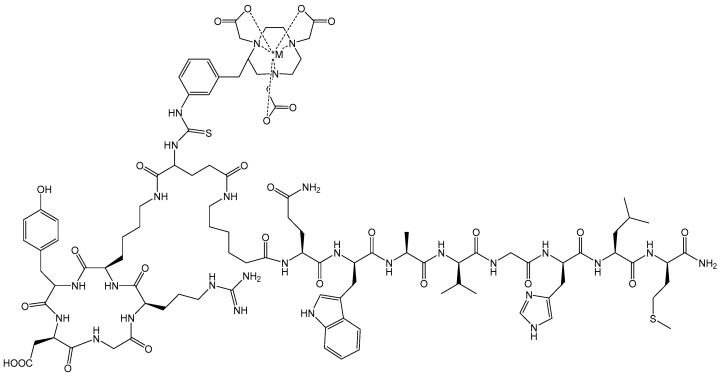
Chemical structures of *M ([^68^Ga-] or [^64^Cu-]) radiolabeled NOTA-RGD-bombesin. RGD-bombesin is a heterodimer of cyclic RGD peptide c(RGDyK) and Aca-bombesin(7–14) through a glutamate linker, with RGD attached to α-carboxylate and bombesin attached to γ-carboxylate.

α_v_β_3_ integrin is often expressed in tandem with the GRPR on the surfaces of most prostate cancers. The α_v_β_3_ integrin has been shown to be successfully targeted using moieties based upon RGD (Arg-Gly-Asp). Taking advantage of the unique opportunity to target more than one biomarker on the surfaces of prostate cancer, Liu and co-workers have reported the synthesis of heterodimeric regulatory peptide probes of RGD/BBN conjugates for GRPR/α_v_β_3_ dual receptor imaging, in which they focused upon PC-3 tumor uptake in nude mice with [^68^Ga-] or [^64^Cu]-radiolabeled [NOTA-RGD-BBN(7-14)NH_2_] (Figure 6), where the RGD and BBN targeting motifs are linked by a glutamic acid [64,65]. In these studies, they observed improved retention of ^64^Cu tracer at 20 h p.i. as compared to the monomeric BBN or RGD conjugates [64]. PC-3 tumor retention at the 20 h time point was 2.04 ± 0.35, 0.44 ± 0.39, and 0.55 ± 0.32% ID/g for [[^67^Cu]Cu-NOTA-RGD-BBN(7-14)NH_2_], [[^67^Cu]Cu-NOTA-BBN(7-14)NH_2_], and [[^67^Cu]Cu-NOTA-RGD] [64].

**Figure 7:**
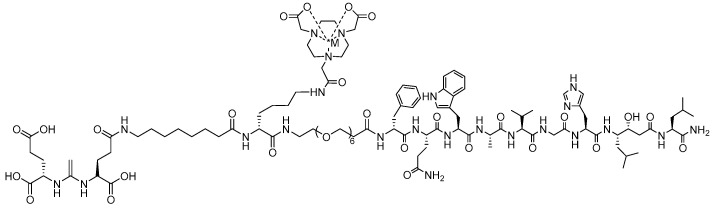
The chemical structure of *M ([^111^In-] or [^68^Ga-]) radiolabeled [DUPA-6-Ahx-(NODAGA)-5-Ava-BBN(7-14)NH_2_], a bivalent targeting vector designed to bind two distinct prostate cancer biomarkers, GRPR and PSMA.

Following the successes of GRPR/α_v_β_3_ dual receptor-targeting radiotracers, investigators have also begun to evaluate heterobivalent GRPR/PSMA-targeting peptides for PCa (Figure 7) [66,67,68,69]. These studies have shown a high propensity for targeting both PSMA and GRPR biomarkers in vivo with high uptake and retention in tumor tissue. Small animal PET and SPECT imaging showed very high uptake in tumors with little or no background radiation in surrounding collateral tissues in the majority of cases. Mitran et al. [68] have studied [^111^In]- and [^68^Ga]-radiolabeled [NOTA-DUPA-RM26] and have evaluated this new class of hybrid radiotracer for stability and efficacy in a prostate cancer model. The new GRPR-/PSMA- heterodimer was radiolabeled with >98% yield and showed very high stability in excess of both EDTA and human serum. In vivo validation of the new radiotracers was completed using PC-3-PIP (GRPR+/PSMA+) xenografted nude mice. Favorable biodistribution of conjugates was demonstrated for both [^68^Ga]- and [^111^In]-NOTA constructs, each exhibiting fast renal clearance and low blood retention with little collateral, normal-tissue accumulation. Tumor tissue uptake at 1 h p.i. was 12 ± 2% ID/g for [[^111^In]In-NOTA-DUPA-RM26] and 8 ± 2% ID/g for [[^68^Ga]Ga-NOTA-DUPA-RM26]. Investigations where co-injection of a respective monomeric ligand was performed significantly decreased the tumor accumulation rate. In vivo small animal imaging in tumor-bearing mice validated biodistribution investigations. Small animal PET images for the [^68^Ga] heterodimer showed very clear tumor visualization. According to the authors, small animal SPECT images for the [^111^In]-radiolabeled hybrid molecule provided higher contrast images when compared to the [^68^Ga]-radiolabeled heterodimer [68].

**Figure 8:**
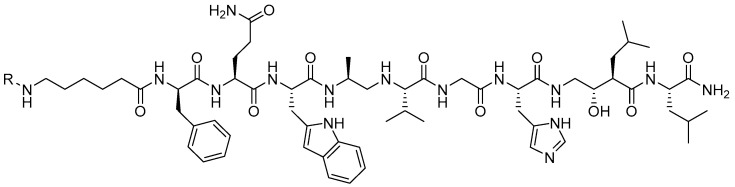
The chemical structure of the GRPR-targeting antagonist peptide (DPhe-Gln-Trp-Ala-Val-Gly-His-Sta-Leu-NH_2_) where the fac-[*M*(CO)_3_(*R*)]^+^ (where R = NOTA or NODAGA and M = [^99m^Tc/^186^Re(CO)_3_]^+^) is conjugated to the N-terminal of the 6-aminohexanoic acid peptide liker via an amide bond.

Makris et al. have also evaluated the unique ability of NOTA and NODAGA to form stable complexes with [^99m^Tc/^186^Re]Tc/Re via the usage of [^*^M(H_2_O)_3_(CO)_3_]^+^ tricarbonyl radiochemistry to produce conjugates that effectively target the GRPR [70]. The inherent ability of NOTA or NODAGA to stabilize the metal center by tridentate, facial co-ordination ([fac-M(CO)_3_]) on the three inherent nitrogen atoms has only been studied minimally. In these studies, the investigators once again utilized the GRPR-targeting peptide antagonist (DPhe-Gln-Trp-Ala-Val-Gly-His-Sta-Leu-NH_2_) linked to the metal complex via a 6-aminohexanoic acid pharmacokinetic modifier (Figure 8) [70]. Both NOTA and NODAGA derivatives showed very high in vitro stability and high GRPR-binding affinity with IC_50_s in the single-digit nanomolar range (2–3 nM). In vivo biodistributions were conducted in PC-3 tumor-bearing ICR SCID mice, where in this instance, the NODAGA derivative demonstrated superior tumor uptake (8.3 ± 0.9% ID/g) at 1 h p.i., with rapid renal-urinary excretion and high-quality, high-contrast SPECT/CT images. The NOTA derivative, however, showed good tumor accumulation of 4.3 ± 0.7% ID/g at 1 h [70]. Both derivatives demonstrated about 82–96% loss of their tumor uptake in blocking studies using unlabeled peptide, proving the GRPR-mediated uptake of radiotracer [70].

**Figure 9:**
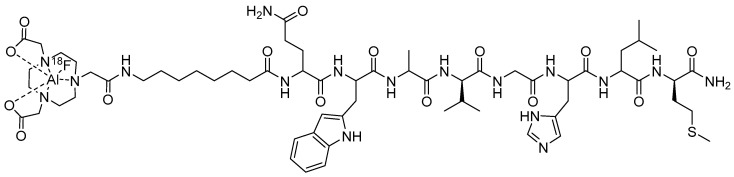
Chemical structure of a NOTA-conjugated BBN analog [NOTA-8-Aoc-BBN(7-14)NH_2_] radiolabeled with Al[^18^F]F.

Perhaps one of the more exciting radiolabeling strategies in the last 50 years is that of using the Al[^18^F]F method to complex [^18^F] to biologically active cell-targeting moieties [29]. In this method, researchers have utilized the inherent coordinating ability of the nitrogen atoms of NOTA to complex the Al[^18^F]F^2+^ cation. Dijkgraaf and co-workers have produced [Al[^18^F]F-NOTA-8-Aoc-BBN(7-14)NH_2_] (Figure 9) as a potential GRPR PET-imaging agent [71]. In this study, the radiosynthesis of [Al[^18^F]F-NOTA-8-Aoc-BBN(7-14)NH_2_] was efficient, and the product was purified using reversed-phase HPLC. In a competitive binding assay using nonradioactive [Al[^nat^F]F-NOTA-8-Aoc-BBN(7-14)NH_2_], a very high binding affinity for the GRPR expressed on the surfaces of PC-3 cells (IC_50_ of 0.28 ± 0.15 nM) was observed. In vivo studies conducted in male nude BALB/c mice bearing subcutaneous PC-3 tumors showed tumor accumulation values of 2.15 ± 0.55% ID/g at 1 h p.i. Tumor uptake was effectively reduced by co-administration of macroscopic amounts of [NOTA-8-Aoc-BBN(7-14)NH_2_], confirming receptor-mediated uptake of the radioligand for the GRPR [71]. The renal-urinary excretion pathway was the primary route of excretion for this agent despite the production of an overall neutral metal-NOTA complex. Small animal PET/CT produced high-quality, high-contrast images with clear tumor accumulation of the [Al[^nat^F]F-NOTA-8-Aoc-BBN(7-14)NH_2_] conjugate [71]. In the same study, the authors reported uptake of [[^68^Ga]Ga-NOTA-8-Aoc-BBN(7-14)NH_2_] radiotracer in GRPR-expressing PC-3 tumors to be 1.24 ± 0.26% ID/g at 1 h p.i., suggesting the superiority of using the Al[^18^F]F method of radiosynthesis to produce clinically useful cell-targeting agents for PET imaging of GRPR-expressing tumors.

### 3.2. Somatostatin Receptor Subtype 2 (SSTR2)-Targeting Radioligands

Somatostatin (SST) cell-targeting radiotracers have been the hallmark for development of new theranostic radiotracers for more than three decades. SST is a neuropeptide hormone that is expressed largely in the central and peripheral nervous system. The most clinically useful subtype, SSTR2, is expressed in very high levels on human neuroendocrine tumors (NETs) and neuroendocrine carcinomas (NECs) [72,73,74]. The elevated levels of SST receptors on NE neoplasms, as well as SST’s antiangiogenic effects, has led to the development of radiotheranostic analogs of SST. The evolution of SST-targeting radiotracers began with Lambert’s 1989 study using ^123^I-radiolabeled Try^3^-octreotide [D-Phe-Cys-Phe-D-Trp-Lys-Thr-Cys-Thr(ol)], known as TOC [75], to visualize endocrine-related tumors [76]. These studies led to the development of clinically approved [[^111^In]In-octreotide] (SANDOSTATIN^®^), which has been the benchmark for radiolabeled, peptide-based, cell-targeting agents for many years. However, recent advancements in the development of novel NET-targeting agents to include the usage new radiometal complexing agents and modification of the receptor-specific active site of the peptide have catalyzed research investigations to develop additional NET cell-targeting agents to include DOTA-TOC and DOTA-TATE [77,78]. Recent FDA approval of [[^177^Lu]Lu-DOTATATE] (LUTATHERA^®^) and [[^68^Ga]Ga-DOTATATE] (NETSPOT^®^) for the treatment and diagnosis of NETs has marked a significant milestone in the field of radiotheranostics [79]. As a result, a successful theranostic approach targeting these receptors has emerged and has shown positive results in reducing tumor growth and improving patient survival. The success of SST-targeted peptide-based agents has paved the way for exploring similar approaches for targeting cell-surface receptors and the development of novel radioligands via other peptide-based agents including the Melanocortin-1 receptor (MC1R).

**Figure 10:**
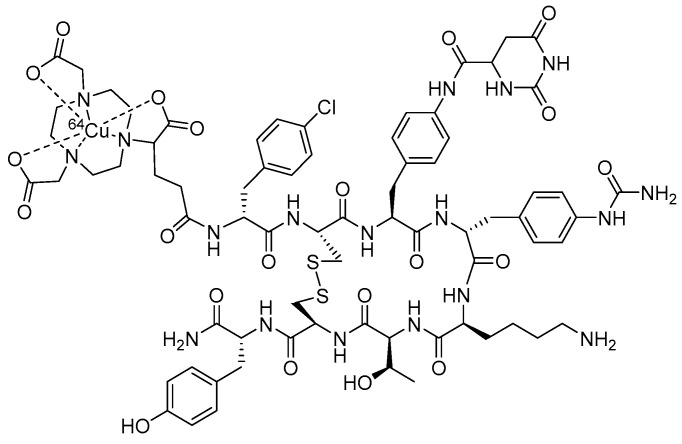
The chemical structure of the [[^64^Cu]Cu-NODAGA-JR11] conjugate. In the related conjugate DOTA-JR11, the DOTA chelator is attached to the N-terminus of JR11.

Among the several chelators used in SSTR2-targeting radiopharmaceuticals, NOTA and its derivatives play a crucial role in chelating various theranostic radioisotopes. This is largely attributed to the chelator’s ability to efficiently form stable complexes with a diverse range of radiotracers. A Switzerland-based research group explored the radiosynthesis of various radiometal complexes for SSTR2 targeting using both NOTA and NODAGA. In one of their detailed reports, Fani et al. successfully conducted a direct comparison of an SSTR-targeting drug antagonist [[^64^Cu]Cu-NODAGA-JR11] and the agonist [[^64^Cu]Cu-DOTA-TATE] determined NODAGA to be favorable both for in vitro and in vivo investigations [80]. Previous investigations of DOTA and TE2A-coupled JR11 demonstrated inferior affinity compared to other JR11 conjugates [81,82]. ^64^Cu radiolabeling of NODAGA-JR11 (40 MBq/nmol, 99%, Figure 10) and DOTA-TATE (7 MBq/nmol, 97%) were prepared at 95 °C for 10 min. To determine SSTR affinity, HEK-293 human kidney cells were used in a saturation binding assay in which K_d_ values were determined to be 5.7 ± 0.95 nM and 20.1 ± 4.4 nM for [[^64^Cu]Cu-NODAGA-JR11] and [[^64^Cu]Cu-DOTA-TATE], respectively. Subsequently, HEK-293 xenografted nude mice were utilized for in vivo and PET/CT imaging investigations. Over various time points, [^64^Cu]Cu-NODAGA-JR11] demonstrated favorable pharmacokinetics in comparison to [[^64^Cu]Cu-DOTA-TATE] with fast blood clearance and elimination through the kidneys. [[^64^Cu]Cu-NODAGA-JR11] accumulated in SSTR2 positive organs and remained stable in the tumor between 1 and 4 h p.i. (20.6 ± 3.7–19.0 ± 3.1% ID/g) [80]. A high tumor-to-background ratio of 192.5 was also noted at 24 h p.i for the NODAGA derivative. Despite a similar tumor uptake of [[^64^Cu]Cu-DOTA-TATE], slower blood and heart clearance, along with significantly higher uptake in SSTR2-negative organs, was observed. Overall, [[^64^Cu]Cu-DOTA-TATE] had lower tumor-to-muscle ratios in comparison to the NODAGA derivative up to the 24 h time point. These in vivo findings show the instability of the ^64^Cu-labeled DOTA derivative, reinforcing the superior chelation stability of [[^64^Cu]Cu-NODAGA-JR11], which is better suited for clinical and preclinical validation.

**Figure 11:**
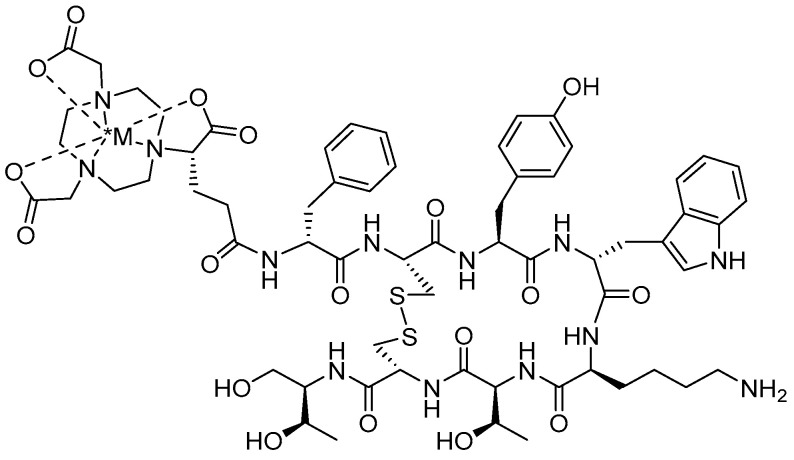
Chemical structure of *M ([^111^In-], [^67^Ga-], or [^90^Y-]) radiolabeled [NODAGA-Tyr^3^-Octreotide], designated as NODAGATOC.

Building on their previous work, the same research group elucidated the use of NODAGA as an alternative chelator to DOTATOC for radiolabeling with [^67^Ga], [^111^In], and [^90^Y] in SSTR2-targeted imaging and therapy. This comparison stemmed from concerns that DOTATOC exhibited limited spatial clearance between the pharmacophore and BFCA, potentially causing steric strain that could impact receptor binding and overall pharmacokinetics. To address this limitation, a monoreactive NOTA-derived chelator, NODAGA, was conjugated to octreotide at the tyrosine-3 (Tyr^3^) position, facilitating preclinical evaluation for selective targeting of SSTR2 (Figure 11) [83]. NOTA was selected due to its well-documented ability to form highly stable complexes with Ga(III) and In(III), ensuring robust radiolabeling and in vivo stability. NODAGA-Tyr^3^-octreotide (NODAGATOC) was efficiently radiolabeled with both [^67^Ga] and [^111^In]. The NODAGA complexed agents remained stable in excess DTPA, human serum, and rat liver homogenate with no degradation of the compound. IC_50_ of [[^67^Ga]Ga-NODAGATOC] and [[^111^In]In-NODAGATOC] were 3.5 ± 1.6 and 1.7 ± 0.2 nM in a SSTR2 receptor expressing cell line, which demonstrates improved performance in comparison to [[^68^Ga]Ga-DOTATOC] (2.5 ± 0.5 nM) [83]. To determine in vivo affinity, rat pancreas tumor cell line AR4-2J (SSTR+) was inoculated in female nude mice to determine pharmacokinetic ability via biodistribution investigations. A direct in vivo comparison was made between the superior [[^67^Ga]Ga/[^111^In]In-NODAGATOC] and [[^111^In]In-DOTATOC], with both compounds demonstrating rapid clearance from the blood and primary excretion through the renal-urinary system. [[^67^Ga]Ga-NODAGATOC] exhibited higher uptake in all SSTR-positive tissues, with a tumor uptake at 21.2 ± 3.5% ID/g at 4 h p.i. In contrast, [[^111^In]In-DOTATOC] showed a lower accumulation in the tumor at 4 h p.i. of 12.50 ± 0.65% ID/g [83]. Tumor to kidney ratios indicated that the NODAGA conjugate had a threefold improvement over the DOTA conjugate at 4 h p.i., with this advantage increasing to nearly sixfold at 24 h p.i. Additional studies completed with [[^111^In]In-DTPA-octreotide] in identical tumor models showed tumor accumulation of only 3.03 ± 0.26% ID/g at 4 h p.i. [84], further confirming the NODAGA complexed superiority for SSTR targeting radiopharmaceuticals.

More recently, another research group from the Netherlands explored the use of NOTA as a chelator for SSTR2 targeting. Laverman et al. [85] synthesized a NOTA-octreotide analog, NOTA-D-Phe-cyclo[Cys-Phe-D-Trp-Lys-Thr-Cys]-Thro, which was subsequently radiolabeled with Al[^18^F]F and [^68^Ga]Ga. Both radiotracers remained stable in human serum and had favorable IC_50_ values of 3.6 ± 0.6nM and 13 ± 3nM for Al[^18^F]F and [^68^Ga]Ga, respectively. Studies demonstrating in vivo tumor uptake of the NOTA-octreotide analog exhibited results of 29.2 ± 0.5% ID/g at 2 h p.i. for [^68^Ga]Ga and 28.3 ± 5.7% ID/g for Al[^18^F]F [85]. Both radiotracers demonstrated receptor-mediated uptake in the presence of a co-blocking injection, with minimal to no uptake observed in normal SSTR-negative tissues. These findings support the use of NOTA-conjugated SSTR agents, showing improved tumor uptake and pharmacokinetics for potential clinical applications.

**Figure 12:**
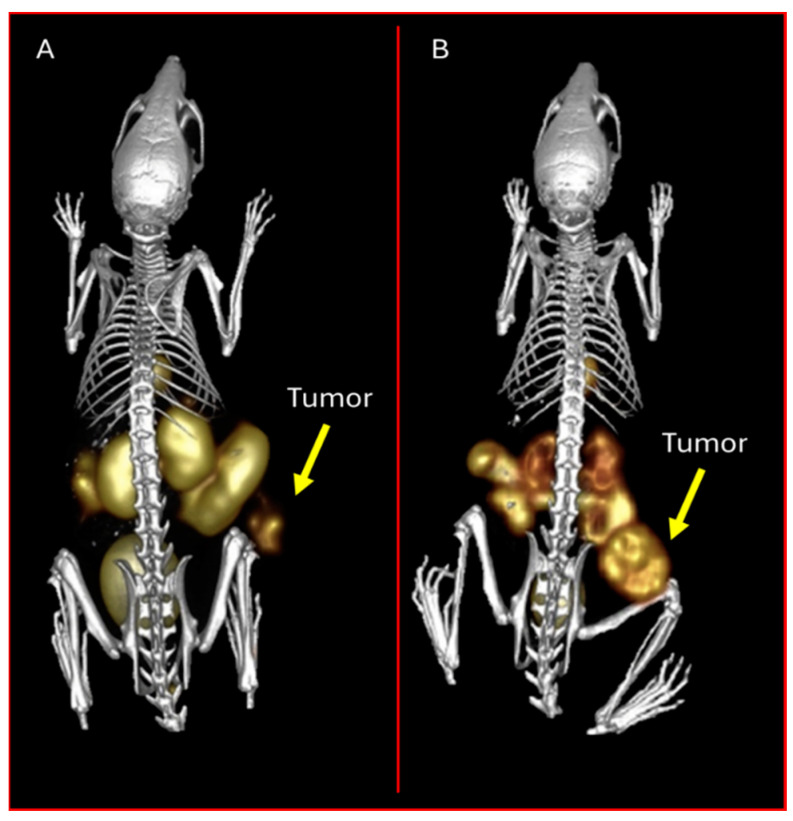
Micro-SPECT/CT images taken at 1 h p.i. in mice bearing AR42J tumors on the right hind flank (7.4 MBq). (**A**) [[^99m^Tc][Tc(OH_2_)_3_(CO)_3_]-NODAGA-SST2-ANT] and (**B**) [[^99m^Tc][Tc(OH_2_)_3_(CO)_3_]-NOTA-SST2-ANT]. The tumors (T) are indicated by the yellow arrows. The figure was adapted and modified with permission from reference [86].

The research group at the University of Missouri has contributed to the evaluation of NOTA and NODAGA as optimal bifunctional chelators for [^99m^Tc/^186^Re]Tc/Re via the tricarbonyl labeling methodology to target the somatostatin receptor [86,87]. Makris et al. evaluated [[^99m^Tc][Tc(OH_2_)_3_(CO)_3_]-NOTA-SST2-ANT] and [[^99m^Tc][Tc(OH_2_)_3_(CO)_3_]-NODAGA-SST2-ANT] in vitro and in vivo. These novel agents were synthesized and evaluated in a CF-1 xenografted mice model for their targeting ability and in vivo stability. These organometallic complexes demonstrated high radiochemical yield and in vitro stability. Receptor binding cell assays were conducted to determine the IC_50_ value of [[Re]Re(OH_2_)_3_(CO)_3_]-NODAGA-SST2-ANT] for SSTRs in AR42J rat pancreatic cancer cells. These studies showed an IC_50_ = 91 nM for [[Re]Re(OH_2_)_3_(CO)_3_]-NODAGA-SST2-ANT]. In biodistribution investigations, both agents exhibited favorable pharmacokinetics including rapid blood clearance and fast renal-urinary excretion, with 82% of the injected dose being excreted via the urine within 1 h. Kidney retention was minimal, and no nonspecific accumulation was observed in other organs [87]. In ICR SCID mice, [[^99m^Tc][Tc(OH_2_)_3_(CO)_3_]-NOTA-SST2-ANT] showed tumor uptake of 2.78 ± 0.27% ID/g at 1 h p.i., while [[^99m^Tc][Tc(OH_2_)_3_(CO)_3_]-NODAGA-SST2-ANT] showed higher tumor accumulation of 16.70 ± 3.32% ID/g 1 h p.i. (Figure 12) [86].

Given the promising results of the SSTR as an effective target for radiotherapeutic agents, recent studies have focused on developing new SSTR agonists and antagonists with enhanced site specificity. For example, Liang et al. investigated a novel SSTR antagonist, Asp_2_-PEG_2_-JR11, as a targeting vector, coupled with the NOTA chelator and radiolabeled with Al[^18^F]F [88]. [Al[^18^F]F-NOTA-Asp_2_-PEG_2_-JR11] was prepared in high radiochemical purity. This agent demonstrated high receptor binding affinity in AR42J (SSTR+) cells with an IC_50_ value of 13.18 ± 1.52 nM. In vivo evaluation in mice bearing xenografted AR42J or HCT116 (SSTR2-) tumors showed a tumor uptake of 7.20 ± 0.74% ID/g at 1 h p.i., which was superior to [[^68^Ga]Ga-DOTA-TATE] showing an accumulation of 5.75 ± 0.55% ID/g. At the same time point, blocking studies and small animal PET/CT imaging confirmed the tumor selectivity and targeting efficiency of the new NOTA-conjugated radiotracers, highlighting their potential for improved clinical applications in SSTR-targeted therapies.

### 3.3. Melanocortin-1 Receptor (MC1R)-Targeting Radioligands

Malignant melanoma is the most deadly form of skin cancer with approximately 100,000 new cases annually [89]. Unfortunately, only 35% of melanoma patients can reach the milestone of 5-year survival [90]. The latest cancer statistics predict that more than 8200 deaths occurred in 2024 in the United States [89]. The Melanocortin-1 receptor (MC1R) is an attractive G protein-coupled receptor (GPCR) for melanoma targeting because of its high expression on both human and mouse melanoma samples [91,92,93]. Native α-melanocyte-stimulating hormone (α-MSH) is a linear peptide (Ac-Ser^1^-Tyr^2^-Ser^3^-Met^4^-Glu^5^-His^6^-Phe^7^-Arg^8^-Trp^9^-Gly^10^-Lys^11^-Pro^12^-Val^13^-NH_2_) with the His^6^-Phe^7^-Arg^8^-Trp^9^ targeting vector serving as the MC1R-binding moiety. To overcome the in vivo proteolytic degradation of native α-MSH, non-natural Norleucine^4^ (Nle^4^) and D-Phe^7^ have been introduced to yield NDP-MSH while maintaining sub-nanomolar MC1R-binding affinity [94]. Moreover, the cyclization of α-MSH peptides via disulfide bonds, site-specific metal introduction, and the lactam bridge have also been employed to improve the MC1R binding affinity and to enhance the melanoma-targeting properties of radiolabeled cyclic α-MSH peptides [95]. Among these promising radiolabeled cyclic α-MSH peptides, [[^68^Ga]Ga-1,4,7,10-tetraazacyclododecane-1,4,7,10-tetraacetic acid-Gly-Gly-Nle-c[Asp-His-DPhe-Arg-Trp-Lys]-CONH_2_] ([[^68^Ga]Ga-DOTA-GGNle-CycMSH_hex_]) has been able to produce remarkable PET images of melanoma metastases in the brain, lung, connective tissue, and intestines of melanoma patients by targeting MC1Rs [93], demonstrating the clinical potential of MC1R-targeted peptide radiopharmaceuticals for melanoma imaging and therapy.

**Figure 13:**
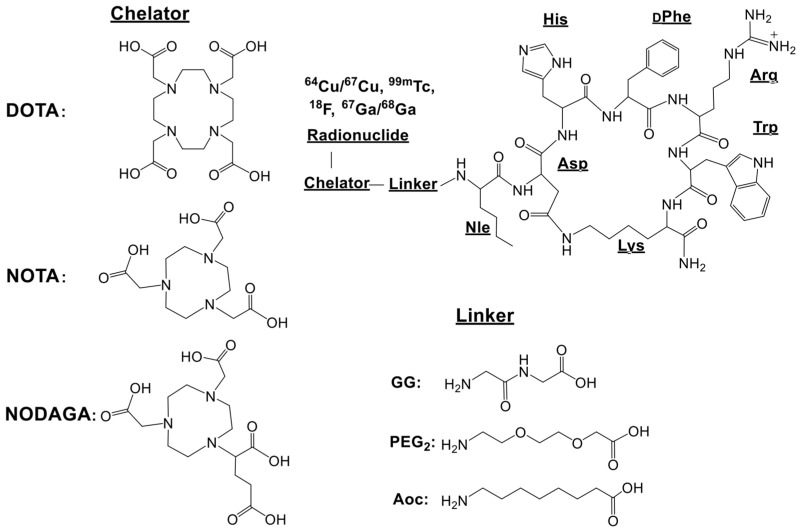
Schematic representation of MC1R-targeted DOTA/NOTA/NODAGA-conjugated CycMSH_hex_ with different linkers and radionuclides.

Copper-64 continues to be at the forefront of nuclear medicine as an attractive radionuclide for clinical PET imaging [4]. It is known that the [^64^Cu]Cu-DOTA metal complex is only moderately stable in vivo due to demetallation, which generally yields high accumulation in non-target tissues such as liver. A biodistribution comparison between [^64^Cu]Cu-DOTA- and [^64^Cu]Cu-NOTA-conjugated to an 8-Aoc-BBN(7-14)NH_2_ GRPR targeting moiety, as previously discussed, has demonstrated NOTA to be a more suitable complex as compared to DOTA in terms of minimizing the demetallation of [^64^Cu]Cu in vivo in a PC-3 tumor-bearing mouse model [17]. In that study, [[^64^Cu]Cu-NOTA-8-Aoc-BBN(7-14)NH_2_] exhibited significantly lower liver accumulation as compared to [[^64^Cu]Cu-DOTA-8-Aoc-BBN(7-14)NH_2_] [17]. This interesting finding on BBN peptides stimulated research efforts around the globe for using [^64^Cu]Cu-NOTA on other GPCR-targeting peptides to include MC1R-targeted α-MSH peptides (Figure 13).

Our very own research group has investigated lactam bridge-cyclized α-MSH peptides for PET imaging of melanoma. Interestingly, initial studies showed NOTA-GGNle-CycMSH_hex_ and DOTA-GGNle-CycMSH_hex_ to display very similar MC1R binding affinities as (1.6 vs. 2.1 nM) on B16/F1 melanoma cells. However, the melanoma targeting and biodistribution properties were dramatically different between [[^64^Cu]Cu-DOTA-GGNle-CycMSH_hex_] and [[^64^Cu]Cu-NOTA-GGNle-CycMSH_hex_] in B16/F1 melanoma-bearing C57 mice [96]. In these studies, the tumor uptake of [[^64^Cu]Cu-NOTA-GGNle-CycMSH_hex_] (12.51 ± 0.44, 12.39 ± 1.61, 12.71 ± 2.68% ID/g) was 2.2, 2.4, and 2.4 times than the tumor uptake for [[^64^Cu]Cu-DOTA-GGNle-CycMSH_hex_] (5.61 ± 0.91, 5.20 ± 1.28, 5.25 ± 1.22% ID/g) at 0.5, 2, and 4 h post-intravenous injection, respectively. Furthermore, the replacement of DOTA with NOTA dramatically decreased the liver accumulation of [[^64^Cu]Cu-NOTA-GGNle-CycMSH_hex_] by 14-fold to 0.69 ± 0.06% ID/g as compared to [[^64^Cu]-DOTA-GGNle-CycMSH_hex_] (10.33 ± 1.18% ID/g) at the 2 h time point_._ Liver retention for [[^64^Cu]Cu-NOTA-GGNle-CycMSH_hex_] was less than 0.8%ID/g at the 4 h and 24 h time points [96]. Liver accumulation and retention for [[^64^Cu]Cu-NOTA-GGNle-CycMSH_hex_] (1.10 ± 0.18, 0.69 ± 0.06, 0.75 ± 0.17, 0.53 ± 0.07% ID/g) was approximately 6.7% to 9.5% of the liver uptake for [[^64^Cu]Cu-DOTA-GGNle-CycMSH_hex_] (11.59 ± 1.77, 10.33 ± 1.18, 9.61 ± 1.34, 7.23 ± 0.58% ID/g) at 0.5, 2, 4, and 24 h post-injection, respectively [96]. The low hepatic uptake and retention of [[^64^Cu]Cu-NOTA-GGNle-CycMSH_hex_], as shown in this study, suggests enhanced in vivo stability of the [^64^Cu]-CuNOTA complex in the [[^64^Cu]Cu-NOTA-GGNle-CycMSH_hex_] peptide, which was consistent with the original findings for [[^64^Cu]Cu-NOTA-8-Aoc-BBN(7-14)NH_2_] [17] and also for [[^64^Cu]Cu-NOTA-STh] peptides which target colorectal lesions [35]. It is worthwhile to note that the substitution of DOTA with NOTA complexing agent also reduced the renal uptake of [[^64^Cu]Cu-NOTA-GGNle-CycMSH_hex_] (9.28 ± 0.77, 4.92 ± 1.43, 3.53 ± 0.57% ID/g) by approximately 35% to 52% at the 0.5, 2, and 4 h time points as compared to [[^64^Cu]Cu-DOTA-GGNle-CycMSH_hex_] (14.44 ± 1.85, 9.30 ± 2.81, 7.45 ± 0.96% ID/g), respectively [96].

**Figure 14:**
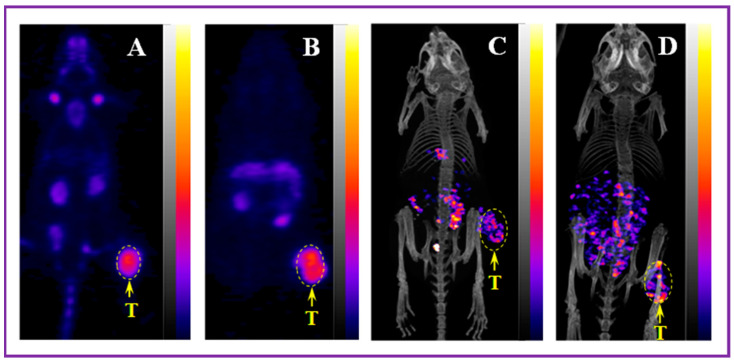
Representative coronal PET images of B16/F10 melanoma-bearing C57 mice using [Al[^18^F]F-NOTA-PEG_2_Nle-CycMSH_hex_] ((**A**), 2 h post-injection) and [[^64^Cu]Cu-NOTA-PEG_2_Nle-CycMSH_hex_] ((**B**), 2 h post-injection) as imaging probes, respectively; representative maximum intensity projection SPECT/CT images of B16/F10 melanoma-bearing C57 mice using [[^99m^Tc]Tc(CO)_3_-NOTA-PEG_2_Nle-CycMSH_hex_] ((**C**), 2 h post-injection) and [[^67^Cu]Cu-NOTA-PEG_2_Nle-CycMSH_hex_] ((**D**), 4 h post-injection) as imaging probes, respectively. Melanoma lesions (T) are highlighted with arrows on images. Figure adapted and modified with permissions from references [97,98,99,100].

In an attempt to examine the effect of the linking moiety on melanoma receptor targeting, our research group has also studied the effects of the GG linker in NOTA-GGNle-CycMSH_hex_ being replaced by polyethylene glycol (PEG_2_) and 8-aminooctanoic acid (Aoc) to generate NOTA-PEG_2_Nle-CycMSH_hex_ and NOTA-AocNle-CycMSH_hex_. Interestingly, NOTA-PEG_2_Nle-CycMSH_hex_ displayed a slightly higher MC1R binding affinity when compared to NOTA-AocNle-CycMSH_hex_ on B16/F10 melanoma cells (1.2 vs. 2.7 nM) [97]. Moreover, the MC1R-specific binding of [[^64^Cu]Cu-NOTA-PEG_2_Nle-CycMSH_hex_] was 2.8 times the binding of [[^64^Cu]Cu-NOTA-AocNle-CycMSH_hex_] for B16/F10 melanoma cells [97]. As anticipated, the melanoma tumor uptake of [[^64^Cu]Cu-NOTA-PEG_2_Nle-CycMSH_hex_] (16.23 ± 0.42, 19.59 ± 1.48, 12.83 ± 1.69, 8.78 ± 2.29% ID/g) was 2.9, 2.5, 2.3, and 5.7 times the uptake of [[^64^Cu]-NOTA-AocNle-CycMSH_hex_] (5.69 ± 0.23, 7.71 ± 0.67, 5.47 ± 0.52, 1.54 ± 0.16% ID/g) at the 0.5, 2, 4, and 24 h time points in B16/F10 melanoma-bearing C57 mice. Both [[^64^Cu]Cu-NOTA-PEG_2_Nle-CycMSH_hex_] and [[^64^Cu]Cu-NOTA-AocNle-CycMSH_hex_] showed comparably low uptake in kidneys (<3.7% ID/g) and liver (<2.2% ID/g) at the 2 h time point, suggesting high in vivo stability for the [^64^Cu]Cu-NOTA complex. Further comparisons of the biodistribution between [[^64^Cu]Cu-NOTA-PEG_2_Nle-CycMSH_hex_] and [[^64^Cu]Cu-NOTA-GGNle-CycMSH_hex_] revealed that accumulation in tumor for [[^64^Cu]Cu-NOTA-PEG_2_Nle-CycMSH_hex_ (19.59 ± 1.48 and 12.83 ± 1.69% ID/g) was 1.6 and 2.1 times the tumor uptake of [[^64^Cu]Cu-NOTA-GGNle-CycMSH_hex_] (7.71 ± 0.67 and 5.47 ± 0.52% ID/g) at 2 h and 4 h post-intravenous injection. Moreover, [[^64^Cu]Cu-NOTA-PEG_2_Nle-CycMSH_hex_] also exhibited higher tumor/kidney ratios when compared to [[^64^Cu]Cu-NOTA-GGNle-CycMSH_hex_] at the 2, 4, and 24 h time points [96,97]. The differences in melanoma uptake and tumor to kidney uptake ratios suggests the profound effects of amino acid, PEG, and hydrocarbon linkers on melanoma targeting and clearance properties of [[^64^Cu]Cu-NOTA-conjugated Nle-CycMSH_hex_ peptides. From a therapeutic perspective, the enhanced tumor/kidney and tumor/liver uptake ratios for [[^64^Cu]Cu-NOTA-PEG_2_Nle-CycMSH_hex_] could minimize the dose absorbed to kidneys and liver while enhancing the therapeutic dose to the tumor in future therapy studies for [[^64^Cu]Cu-NOTA-PEG_2_Nle-CycMSH_hex_] on melanoma (Figure 14).

[^67^Cu], as previously discussed, is the therapeutic, match-paired, surrogate of [^64^Cu] due to their identical coordination chemistry. From a beta decay perspective, [^67^Cu] is a medium-energy (0.562 MeV) β-emitter that is very similar to [^177^Lu] (90.497 MeV). Moreover, as with [^177^Lu], [^67^Cu] is suitable for SPECT scintigraphy due to gamma decay emissions (93 keV, 16% and 185 keV, 49%) that can be used to calculate dosimetry and monitor the therapeutic response. Building on the interesting effects of GG, PEG_2_, and Aoc linkers on melanoma uptake of [[^64^Cu]Cu-NOTA-GG/PEG_2_/AocNle-CycMSH_hex_] [96,97], the melanoma-targeting properties of [[^67^Cu]Cu-NOTA-GGNle-CycMSH_hex_] and [[^67^Cu]Cu-NOTA-PEG_2_Nle-CycMSH_hex_] have also been examined in B16/F10 melanoma-bearing C57 mice to evaluate the usage of [^67^Cu] for the advantage of producing a true matched-pair therapy [98]. In this study, NOTA-PEG_2_Nle-CycMSH_hex_ and NOTA-GGNle-CycMSH_hex_ displayed similar MC1R binding affinities (1.2 vs. 1.6 nM). However, [[^67^Cu]Cu-NOTA-PEG_2_Nle-CycMSH_hex_] displayed higher cellular uptake that was ~2.5x that of [[^67^Cu]Cu-NOTA-GGNle-CycMSH_hex_] in the same MC1R B16/F10 melanoma cell line [98]. Furthermore, [[^67^Cu]Cu-NOTA-PEG_2_Nle-CycMSH_hex_] exhibited higher B16/F10 melanoma uptake than [[^67^Cu]Cu-NOTA-GGNle-CycMSH_hex_]. The tumor accumulations for [[^67^Cu]Cu-NOTA-PEG_2_Nle-CycMSH_hex_] (27.97 ± 1.98, 24.10 ± 1.83, 9.13 ± 1.66% ID/g) was 2.4, 2.7, and 1.9 times the tumor uptake of [[^67^Cu]Cu-NOTA-GGNle-CycMSH_hex_] (11.66 ± 1.94, 8.79 ± 1.31, 4.92 ± 1.58% ID/g) at 2, 4, and 24 h post-intravenous injection, respectively [98]. Therefore, it is important to note that biodistribution studies are necessary to fully appreciate the potential difference in tumor uptake for these peptides which exhibit similar in vitro receptor binding affinities.

[[^67^Cu]Cu-NOTA-PEG_2_Nle-CycMSH_hex_] and [[^67^Cu]Cu-NOTA-GGNle-CycMSH_hex_] showed similar renal and liver uptakes in B16/F10 melanoma-bearing C57 mice. However, [[^67^Cu]Cu-NOTA-PEG_2_Nle-CycMSH_hex_] exhibited higher tumor/kidney and tumor/liver uptake ratios when compared to [[^67^Cu]Cu-NOTA-GGNle-CycMSH_hex_] because of the higher melanoma tumor uptake for [[^67^Cu]Cu-NOTA-PEG_2_Nle-CycMSH_hex_]. The tumor/kidney ratio of [[^67^Cu]Cu-NOTA-PEG_2_Nle-CycMSH_hex_] (4.41, 9.27, 10.14) was 2.4, 2.7, and 1.9 times the tumor/kidney ratio of [[^67^Cu]-NOTA-GGNle-CycMSH_hex_] (2.29, 2.09, 4.13) at the 2, 4, and 24 h time points, respectively. Moreover, the tumor/liver ratio for [[^67^Cu]Cu-NOTA-PEG_2_Nle-CycMSH_hex_] (25.66, 17.09, 10.87) was 3.3, 2.0, and 1.8 times the tumor/liver ratio of [[^67^Cu]Cu-NOTA-GGNle-CycMSH_hex_] (7.83, 8.62, 6.0) at 2, 4, and 24 h post-intravenous injection [98]. From a therapeutic point of view, the improved tumor/kidney and tumor/liver uptake ratios for [[^67^Cu]Cu-NOTA-PEG_2_Nle-CycMSH_hex_] lend impetus to study the therapeutic effects of this novel radiopharmaceutical in melanoma.

[^67^Ga], as previously discussed, is a cyclotron-produced radionuclide that can be used for SPECT scintigraphy. DOTA has the ability to form stable complexes with [^67^Ga]. However, studies have shown that NOTA and NODAGA can also yield kinetically inert, in vivo stable, metal complexes with [^67^Ga] at even lower reaction temperatures as compared to the corresponding DOTA conjugates [101,102]. [[^67^Ga]Ga-DOTA-GGNle-CycMSH_hex_] can be prepared in greater than 85% radiolabeling yield at 75 °C. Radiolabeling yields for [[^67^Ga]Ga-NOTA-GGNle-CycMSH_hex_] have been shown to be 70% and 85% at 25 °C and 37 °C, respectively. Both [[^67^Ga]Ga-DOTA-GGNle-CycMSH_hex_] and [[^67^Ga]Ga-NOTA-GGNle-CycMSH_hex_] can be radiolabeled in greater than 90% yield after 30 min incubation at 75 °C [102]. Biodistribution studies showed that both [[^67^Ga]Ga-NOTA-GGNle-CycMSH_hex_] and [[^67^Ga]Ga-DOTA-GGNle-CycMSH_hex_] displayed comparable high melanoma uptake (25.53 ± 2.22 vs. 25.12 ± 1.03% ID/g) and similar renal accumulation (8.34 ± 3.25 vs. 8.9 ± 1.81% ID/g) at 2 h post-intravenous injection in B16/F1 melanoma-bearing C57 mice [101].

**Figure 15:**
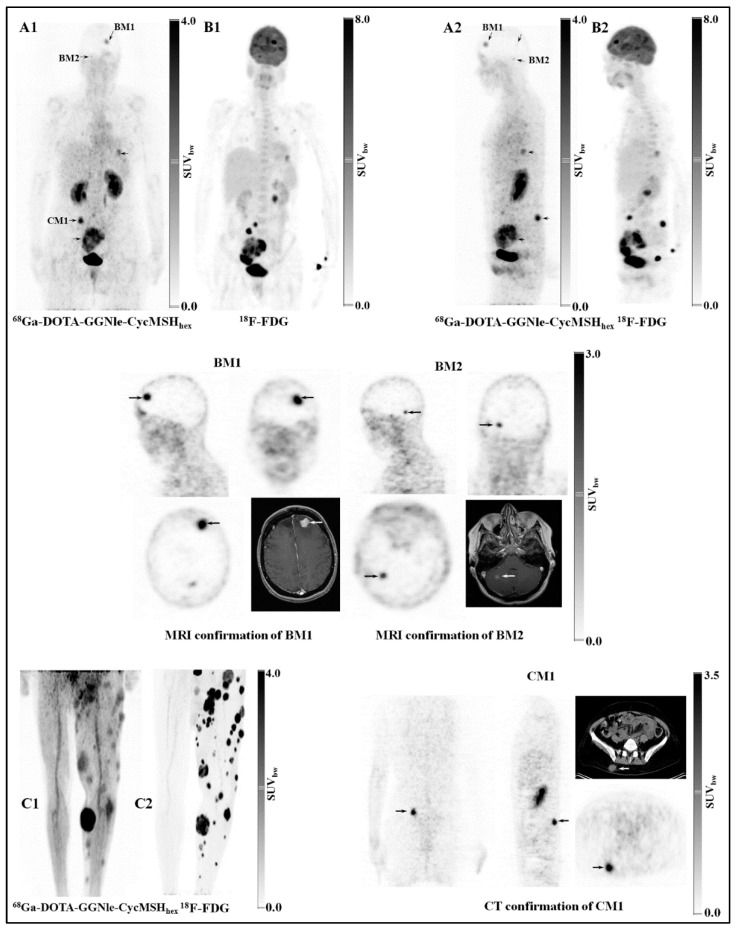
Representative first-in-human PET studies on two melanoma patients. Melanoma metastases in the brain, lung, connective tissue, and small intestine of patient 1 (**A1**,**A2**) were visualized by PET using [[^68^Ga]Ga-DOTA-GGNle-CycMSH_hex_]. Melanoma metastases on the left leg of patient 2 (**C1**) were imaged by PET using [[^68^Ga]Ga-DOTA-GGNle-CycMSH_hex_]. [^18^F]F-FDG PET (**B1**,**B2**,**C2**) studies were performed on the same patients for comparisons. The melanoma metastases in the brain (BM1 and BM2) were confirmed by MRI and the metastasis in the connective tissue (CM1) was confirmed by CT. Melanoma metastases are highlighted with arrows on the images. SUV_bw_, standardized uptake value based on body weight. The figure was adapted and modified with permission from reference [93].

To expand upon the development of novel PET radiotracers for molecular imaging of melanoma, our research team also investigated the ability for [[^68^Ga]Ga-DOTA-GGNle-CycMSH_hex_] to target the MC1R. [^68^Ga] is an attractive PET radionuclide which is readily available from an in-house commercial [^68^Ge]/[^68^Ga] generator. The advantages of using MC1R-targeted [[^68^Ga]Ga-DOTAGGNle-CycMSH_hex_] PET radiotracer include a unique opportunity to harvest the excellent imaging properties of [^68^Ga] for high-sensitivity, tumor-specific imaging of melanoma metastases. In 2018, our research team demonstrated the first-in-human PET study for [[^68^Ga]Ga-DOTA-GGNle-CycMSH_hex_] on melanoma patients, clearly detecting melanoma metastases in the brain, lung, connective tissue, and intestines [93]. The successes of MC1R-targeted [[^68^Ga]Ga-DOTA-GGNle-CycMSH_hex_] on melanoma patients not only demonstrated the clinical relevance of MC1R as a molecular target for human melanoma imaging, but also highlighted the need to develop MC1R-targeted therapeutic peptides for treating patients with melanoma metastases (Figure 15).

[^99m^Tc] continues to be the foundation for SPECT molecular imaging of human tumors and in vivo metabolic processes because of its cost-effectiveness and availability as ^99m^TcO_4_^−^ from an on-site [^99^Mo]/[^99m^Tc] generator. Traditionally, the reduction of [^99m^TcO_4_]^−^ by SnCl_2_ readily yields monooxo ([^99m^Tc=O]^3+^) core complexes for coordination by N and S atoms in a bifunctional chelating agent that is attached to biomolecules. For instance, in vivo stable monooxo complexes of mercaptoacetyltriglycine (MAG_3_)-GGNle-CycMSH_hex_ and Ac-Cys-Gly-Gly-Gly (AcCG_3_)-GGNle-CycMSH_hex_ have been reported for melanoma imaging [103]. [[^99m^Tc]Tc-MAG_3_-GGNle-CycMSH_hex_] and [[^99m^Tc]Tc-AcCG_3_-GGNle-CycMSH_hex_] exhibited 4.64 ± 1.06 and 9.76 ± 4.90% ID/g on B16/F1 melanoma at 2 h post-intravenous injection [103]. Later, the development of [[^99m^Tc]Tc(CO)_3_(OH_2_)_3_]^+^ tricarbonyl, Isolink^®^ kit offered another robust method for [^99m^Tc]Tc-labeling through the replacement of the three labile water molecules with various donor atoms present in a host of bifunctional chelating ligands. The radiolabeling of hydrazinonicotinamide (HYNIC)-GGNle-CycMSH_hex_ with [^99m^Tc] was reported to have been easily achieved by two approaches: (1) via reaction of the peptide conjugate with the [[^99m^Tc]Tc(CO)_3_(OH_2_)_3_]^+^ tricarbonyl intermediate; (2) through EDDA/tricine co-ligands. Interestingly, [[^99m^Tc]Tc-(EDDA)-HYNIC-GGNle-CycMSH_hex_] exhibited much higher B16/F1 tumor uptake (14.14 ± 4.90% ID/g) than that of [[^99m^Tc]Tc-(CO)_3_-HYNIC-GGNle-CycMSH_hex_] (5.84 ± 1.26% ID/g) at the 2 h time point [103]. Meanwhile, the liver and kidney accumulations of [[^99m^Tc]Tc-(EDDA)-HYNIC-GGNle-CycMSH_hex_] (0.52 ± 0.05 and 7.52 ± 0.96% ID/g) were only 1.4% and 42.5% of the liver and kidney uptakes of [[^99m^Tc]-(CO)_3_-HYNIC-GGNle-CycMSH_hex_] (38.11 ± 2.31 and 17.69 ± 4.06% ID/g) at 2 h post-intravenous injection [103]. Clearly, the difference in the coordination of the [^99m^Tc] core affected the uptake in tumor, liver, and kidney of [^99m^Tc]Tc-labeled lactam-cyclized α-MSH peptides in melanoma-bearing mice. Building on the successes of [[^99m^Tc]Tc-(EDDA)-HYNIC-GGNle-CycMSH_hex_], linker optimization yielded [[^99m^Tc]Tc-(EDDA)-HYNIC-AocNle-CycMSH_hex_] showing higher tumor uptake and tumor/kidney ratios than [[^99m^Tc]Tc-(EDDA)-HYNIC-GGNle-CycMSH_hex_] [104].

Research studies to evaluate the effects of metal complexing agents such as NOTA- and NODAGA-conjugated α-MSH peptides for melanoma targeting and clearance have also been investigated by our research team considering the ability of tridentate NOTA and NODAGA to coordinate [^99m^Tc(CO)_3_]^+^ tricarbonyl core in a facial fashion via three N atoms [105]. [[^99m^Tc]Tc-(CO)_3_-NOTA-GGNle-CycMSH_hex_] (19.76 ± 3.62% ID/g) displayed 1.7 times higher B16/F10 melanoma uptake than [[^99m^Tc]Tc-(CO)_3_-NODAGA-GGNle-CycMSH_hex_] (7.19 ± 1.80% ID/g), whereas the renal uptake of [[^99m^Tc]Tc-(CO)_3_-NOTA-GGNle-CycMSH_hex_] (1.59 ± 0.52% ID/g) was only 18% of that of [[^99m^Tc]Tc-(CO)_3_-NODAGA-GGNle-CycMSH_hex_] (8.66 ± 2.59% ID/g) at 2 h post-intravenous injection [105]. Biodistribution studies revealed that the tumor uptake of [[^99m^Tc]Tc-(CO)_3_-NOTA-GGNle-CycMSH_hex_] (19.76 ± 3.62% ID/g) was 3.4 times higher than the tumor accumulation of [[^99m^Tc]Tc-(CO)_3_-HYNIC-GGNle-CycMSH_hex_] (5.84 ± 1.26% ID/g), whereas the renal and liver uptake of [[^99m^Tc]Tc-(CO)_3_-NOTA-GGNle-CycMSH_hex_] (1.59 ± 0.52 and 1.57 ± 0.32% ID/g) was only 9% and 4% of that of [[^99m^Tc]Tc-(CO)_3_-HYNIC-GGNle-CycMSH_hex_] (17.69 ± 4.06 and 38.11 ± 2.31% ID/g) at the 2 h time point, respectively. Clearly, a change from HYNIC to NOTA chelating agent dramatically reduced the renal and liver accumulation of tracer and enhanced the tumor uptake of [[^99m^Tc]Tc-(CO)_3_-NOTA-GGNle-CycMSH_hex_] when compared to [[^99m^Tc]Tc-(CO)_3_-HYNIC-GGNle-CycMSH_hex_]. A detailed comparison of the biodistribution between [[^99m^Tc]Tc-(CO)_3_-NOTA-GGNle-CycMSH_hex_] and [[^99m^Tc]Tc(EDDA)-HYNIC-GGNle-CycMSH_hex_] demonstrated higher tumor uptake and lower renal accumulation for [[^99m^Tc]Tc-(CO)_3_-NOTA-GGNle-CycMSH_hex_] in melanoma-bearing mice. The tumor uptake of [[^99m^Tc]Tc-(CO)_3_-NOTA-GGNle-CycMSH_hex_] (19.76 ± 3.62% ID/g) was 40% higher than that of [[^99m^Tc]Tc(EDDA)-HYNIC-GGNle-CycMSH_hex_] (14.14 ± 4.90% ID/g), whereas the renal accumulation of [[^99m^Tc]Tc-(CO)_3_-NOTA-GGNle-CycMSH_hex_] (1.59 ± 0.52% ID/g) was only 21% of the renal uptake of [[^99m^Tc]Tc(EDDA)-HYNIC-GGNle-CycMSH_hex_] (7.52 ± 0.96% ID/g) at the 2 h time point [105].

Building on the promising melanoma targeting properties of [[^99m^Tc]Tc-(CO)_3_-NOTA-GGNle-CycMSH_hex_] (tumor, 19.76 ± 3.62% ID/g; kidneys, 1.59 ± 0.52% ID/g, 2 h post-intravenous injection), the effects of PEG_2_ and Aoc linkers on melanoma uptake was further investigated in B16/F10 melanoma-bearing C57 mice. Interestingly, the tumor uptake of [[^99m^Tc]Tc-(CO)_3_-NOTA-PEG_2_Nle-CycMSH_hex_] (31.93 ± 2.57 and 20.31 ± 3.23% ID/g) was 1.6 and 3.4 times than the tumor accumulation for [[^99m^Tc]Tc-(CO)_3_-NOTA-AocNle-CycMSH_hex_] (19.43 ± 2.95 and 5.89 ± 1.71% ID/g) at 2 h and 4 h post-intravenous injection [99]. Moreover, [[^99m^Tc]Tc-(CO)_3_-NOTA-PEG_2_Nle-CycMSH_hex_] displayed higher tumor uptake as compared to [[^99m^Tc]Tc-(CO)_3_-NOTA-GGNle-CycMSH_hex_]. The tumor uptake for [[^99m^Tc]Tc-(CO)_3_-NOTA-PEG_2_Nle-CycMSH_hex_] (31.93 ± 2.57 and 20.31 ± 3.23% ID/g) was 1.6 and 1.8 times higher than the tumor accumulation of [[^99m^Tc]Tc-(CO)_3_-NOTA-GGNle-CycMSH_hex_] (19.76 ± 3.62 and 11.30 ± 2.81% ID/g) at 2 h and 4 h post-intravenous injection, although they exhibited similar low renal uptake and retention (<1.8% ID/g at 2 h post-intravenous injection) [98]. Overall, low renal accumulation and retention coupled with high melanoma tumor uptake of [[^99m^Tc]Tc-(CO)_3_-NOTA-PEG_2_Nle-CycMSH_hex_] underscores the potential application of [[^188^Re]Re-(CO)_3_-NOTA-PEG_2_Nle-CycMSH_hex_] for melanoma therapy in future investigations.

Building on previous successes and the MC1R-targeting properties of CycMSH_hex_, both [Al[^18^F]F-NOTA-GGNle-CycMSH_hex_] and [[^18^F]FamBF_3_-PipNle-CycMSH_hex_] have also been developed via the Al[^18^F]F radiolabeling approach or ^19^F/^18^F isotopic exchange reaction. The biodistribution and pharmacokinetics of the new [^18^F] conjugates have been evaluated in B16/F10-luc and B16/F10 melanoma-bearing C57 mice [106,107]. Although [Al[^18^F]F-NOTA-GGNle-CycMSH_hex_] and [[^18^F]FamBF_3_-PipNle-CycMSH_hex_] displayed comparable tumor uptake at 1 h post-intravenous injection (7.70 ± 1.71 versus 7.80 ± 1.77% ID/g), the radiochemical yield (RCY) of [Al[^18^F]F-NOTA-GGNle-CycMSH_hex_] reached 94 ± 2.8% [107], whereas [[^18^F]FamBF_3_-PipNle-CycMSH_hex_] only showed 12 ± 6% [107]. Such dramatic differences in radiolabeling efficiency for [^18^F] highlights the practical advantage of the [Al[^18^F]F-NOTA] approach over the [[^18^F]FamBF_3_] method.

Finally, in an attempt to investigate the potential effects of the pharmacokinetic modifying linker on melanoma tumor targeting, both [Al[^18^F]F-NOTA-PEG_2_Nle-CycMSH_hex_] and [Al[^18^F]F-NOTA-AocNle-CycMSH_hex_] were developed by our research team. The new radiotracers showed RCYs between 55% and 66% and were evaluated in B16/F10 melanoma-bearing C57 mice [100]. Both [Al[^18^F]F-NOTA-PEG_2_Nle-CycMSH_hex_] and [Al[^18^F]F-NOTA-AocNle-CycMSH_hex_] exhibited rapid tumor accumulation at 0.5 h post-intravenous injection (12.08 ± 0.79 vs. 15.49 ± 1.51% ID/g). The tumor retention of [Al[^18^F]F-NOTA-AocNle-CycMSH_hex_] was reduced to 12.99 ± 0.98% ID/g at the 1 h time point, whereas uptake in tumor for [Al[^18^F]F-NOTA-PEG_2_Nle-CycMSH_hex_] further increased to 17.44 ± 0.76% ID/g at 1 h post-intravenous injection. Evaluation of the biodistribution study results between [Al[^18^F]F-NOTA-PEG_2_Nle-CycMSH_hex_] and [Al[^18^F]F-NOTA-GGNle-CycMSH_hex_] revealed the advantages of using [Al[^18^F]F-NOTA-PEG_2_Nle-CycMSH_hex_] considering melanoma tumor and renal accumulation in melanoma tumor-bearing mice. The tumor uptake of [Al[^18^F]F-NOTA-PEG_2_Nle-CycMSH_hex_] (12.08 ± 0.79 and 17.44 ± 0.76% ID/g) was 1.8 and 2.3 times higher than the tumor uptake for [Al[^18^F]F-NOTA-GGNle-CycMSH_hex_] (6.69 ± 1,49 and 7.70 ± 1.71% ID/g) at 0.5 and 1 h post-intravenous injection. Renal accumulation of [Al[^18^F]F-NOTA-PEG_2_Nle-CycMSH_hex_] (7.55 ± 1.13 and 2.07 ± 0.43% ID/g) was 89% and 38% of the renal uptake of [Al[^18^F]F-NOTA-GGNle-CycMSH_hex_] (8.46 ± 3.09 and 5.52 ± 0.57% ID/g) at the 0.5 and 1 h time points [100]. Clearly, substitution of the GG linker with PEG_2_ dramatically enhances the melanoma tumor accumulation and substantially improves the tumor to kidney uptake ratios for [Al[^18^F]F-NOTA-PEG_2_Nle-CycMSH_hex_].

## 4. Conclusions

It is very clear from our point of view that NOTA-based complexing agents possess the ability to span the periodic table to produce robust, kinetically inert, metal complexes for the development of clinically useful theranostic agents. We have described the ability to use NOTA or NODAGA chelating ligands, when radiolabeled with [^64^Cu], [^67^Cu], [^68^Ga], [^67^Ga], [^111^In], [^99m^Tc], and [^18^F], to produce novel cell-targeting agents that maintain high affinity and selectivity for the GRPRs, SSTR2s, and the MC1Rs that are expressed on the surfaces of many primary human tumors and their distant metastases. The nature and versatility of using NOTA/NODAGA-based complexing agents for all of the radionuclides described herein deems NOTA a nearly “universal chelator”, and this is a noteworthy title for this robust metal-complexing agent for the preparation of theranostic peptides, small molecules, and other cell-targeting biomolecules.

## Figures and Tables

**Table 1:** Theranostic radioisotopes to produce metabolically stable, kinetically inert radiocomplexes with NOTA/NODAGA complexing agents.

Isotope	Physical Characteristics	Availability	Specific Activity
Copper-64	t_1/2_ = 12.7 h, β^+^—0.65 MeV, β^−^—0.57 MeV	Widely Available, Cyclotron-Produced	High
Copper-67	t_1/2_ = 2.58 d, γ—93 and 185 keV, β^−^—0.562 MeV	Widely Available, Cyclotron-Produced	High
Gallium-68	t_1/2_ = 1.13 h, β^+^—1.899 MeV	Limited, Ge-68/Ga-68 Generator	High
Gallium-67	t_1/2_ = 78.3 h, γ—93, 185, and 300 keV	Limited, Cyclotron-Produced	High
Indium-111	t_1/2_ = 2.80 d, γ—171 and 245 keV	Widely Available, Cyclotron-Produced	High
Technetium-99m	t_1/2_ = 6.04 h, γ—140 keV	Widely Available, Generator-Produced	High
Fluorine-18	t_1/2_ = 110 min, β^+^—0.635 MeV	Widely Available, Cyclotron-Produced	High

## Data Availability

All data are contained in the manuscript.
